# Color Stability of Single-Shade Resin Composites in Direct Restorations: A Systematic Review and Meta-Analysis of Randomized Controlled Trials

**DOI:** 10.3390/polym16152172

**Published:** 2024-07-30

**Authors:** Caroline de Farias Charamba Leal, Samille Biasi Miranda, Everardo Lucena de Alves Neto, Keitry Freitas, Wesley Viana de Sousa, Rodrigo Barros Esteves Lins, Ana Karina Maciel de Andrade, Marcos Antônio Japiassú Resende Montes

**Affiliations:** 1 Departament of Dental Materials, Faculty of Dentistry, University of Pernambuco, Recife 50100-130, PE, Brazil; caroline.charamba@upe.br (C.d.F.C.L.); samille.biasi@upe.br (S.B.M.); w.vianaodontologia@gmail.com (W.V.d.S.); 2 Departament of Restorative Dentistry, Faculty of São Leopoldo de Mandic, Campinas 13045-755, SP, Brazil; dr.everardolucena@gmail.com (E.L.d.A.N.); keitry23@hotmail.com (K.F.); 3 Departament of Restorative Dentistry, School of Dentistry, Federal University of Alagoas, Maceió 57072-900, AL, Brazil; rodrigo.lins@foufal.br; 4 Departament of Restorative Dentistry, Federal University of Paraíba, João Pessoa 58051-900, PB, Brazil; kamandrade@hotmail.com

**Keywords:** composite resins, dental materials, permanent dental restoration, color, color perception, systematic review, randomized controlled trial

## Abstract

The objective was to compare the color match and color stability behavior of single- and multi-shade resin-based composites (RBCs) used for direct restorations. This review was conducted according to the Preferred Reporting Items for Systematic Reviews and Meta-Analyses guidelines. Randomized clinical trials evaluating the shade performance of single-shade RBCs in direct restorations were included. A search of the scientific literature was performed in five databases (April 2024). The meta-analysis was performed using RevMan 5.4, calculating the risk difference (RD) and 95% confidence interval (CI) of the dichotomous outcome using a random effects model. Bias was assessed using the RoB 2.0 tool, and certainty of evidence was assessed using the GRADEpro tool. Four studies were selected, with 263 restorations analyzed. The results showed comparable performance between single-shade RBCs and multi-shade RBCs in terms of color match and color stability over 12 months. Three studies had a low risk of bias with all expected results, and one study had some concerns. The certainty of evidence for color stability was considered low for all follow-up periods due to the small number of events and sample size. According to the United States Public Health Service Evaluation (USPHS) and the World Dental Federation (FDI), there is comparable clinical color performance between single-shade and multi-shade RBCs over 12 months.

## 1. Introduction

Resin-based composites (RBCs) have emerged as the preferred esthetic material for dental restorations in both anterior and posterior teeth [1]. However, achieving color harmony between the composite resin and the adjacent dental substrate poses a significant challenge [2]. The effectiveness of a restoration is closely related to its surface properties and color stability [3]. Given the subjective nature of color matching, accurate shade selection remains a formidable task in recreating the natural appearance of dental elements [4]. The proposed technique for achieving color involves selecting a resin from various types of hues, chroma, and luminosity, as well as different optical properties such as opacity, translucency, and opalescence, which requires time and increased cost [5]. Consequently, there is an urgent need to continuously refine techniques that simplify the process of composite resin color selection, thereby reducing clinical protocols and chair time [6].

According to dentists, tooth color is the second most influential factor in smile preference and, along with tooth arrangement, is one of the first features noticed by patients when they smile [7]. The lighter the tooth color is, the more attractive the smile is [8]. This highlights the importance of achieving the correct shade of resin during the restorative procedure. In addition, the resin must exhibit color stability in the oral cavity; therefore, esthetic restoration failure due to discoloration is a relevant clinical issue [9]. Errors in shade selection and pigmentation are easily noticed by patients, negatively affecting their perception and leading to the need for restoration repair or replacement [10]. Replacement of restorations is costly and carries the risk of sacrificing a healthy tooth structure, which may compromise tooth vitality and accelerate the destructive tooth cycle [11].

Dental materials that can adapt to human tooth color offer numerous advantages, including esthetic enhancement, reduced reliance on shade guides, and reduced risk of shade mismatch [1]. This adaptability, often referred to as the “chameleon effect” [12], has led to the emergence of single-shade RBCs equipped with “smart shade technology” [8]. This “chameleon effect” is made possible by the ability of the resin to absorb light from the surrounding tooth [5]. These materials promise to capture the adjacent coloration of restored dental elements, facilitated by particles that produce red/yellow hues that blend with the surrounding coloration, complemented by uniformly sized spherical particles that adjust the transmitted color [8]. This new ability imparted to resins means that the perceived color of a region shifts toward the color of the surrounding area, eliminating or neutralizing color discrepancies between the tooth and the restoration [5]. In addition, single-shade RBCs streamline the color selection process by eliminating the need to choose between different resin shades, as is the case with multi-shade RBCs, thus reducing the time lost in the clinical resin selection process [13]. These materials are able to match all the shades in the Vita Classic Shade Guide [14], as demonstrated in a previous study that found single-shade resins to have a high color adjustment potential (CAP) in human incisors with various shades [12].

While in vitro studies indicate comparable color matching between single-shade and multi-shade RBCs [12,13], others indicate better color matching with single-shade RBCs [6]. In addition to comparable performance in terms of their ability to match the surrounding tooth substrate, restorations with single-shade RBCs were significantly whiter than those with the original tooth shade [15]. Single-shade RBCs have greater chroma adjustment potential than multi-shade resins, enabling them to match a wide range of human tooth shades. However, this ability may be compromised in deep restorations due to increased translucency and structural coloration, resulting in light reflection, even at longer wavelengths [16]. In addition, there are concerns regarding the color stability of single-shade resins. in vitro studies have shown that they are susceptible to discoloration from commonly consumed beverages such as wine, coffee, and black tea [3,17,18]. Conversely, clinical studies indicate that color stability is comparable to that of multi-shade resins [1,19]. Therefore, there is a need to understand the clinical color behavior of single-shade RBCs in terms of color matching and stability.

Based on current evidence from clinical and laboratory studies, there is a lack of definitive understanding of the favorable clinical color behavior of single-shade RBCs, leading to uncertainties regarding their suitability for clinical use. There is limited evidence for the in vivo color stability of single-shade RBCs; therefore, there is an urgent need for an evidence synthesis study that merges data from multiple primary clinical investigations. Such an effort aims to provide robust and reliable scientific evidence on the color matching and color stability of this novel material. This initiative aims to provide stronger scientific support for the color match of this innovative restorative material and to either confirm or challenge its suitability for use. The aim of this systematic review was to compare the clinical color matching and stability of single- and multi-shade RBCs in direct restorations.

## 2. Materials and Methods

### 2.1. Protocol and Registration

This systematic review was conducted following the guidelines of the PRISMA (Preferred Reporting Items for Systematic Reviews and Meta-Analyses) guidelines [20] and was structured as follows: (1) identification of the guiding question, (2) collection of relevant studies, (3) determination of inclusion and exclusion criteria, (4) data extraction, and (5) synthesis of the results [21]. Prior to commencement, the methodology of this study was registered in PROSPERO (International Prospective Register of Systematic Reviews) with the protocol number CRD42024529670.

### 2.2. Eligibility Criteria

The guiding question for this review was “Do single-shade RBCs have shade performance comparable to that of multi-shade resin composites in direct restorations?” The population/problem, intervention/exposure, comparison, and outcome of the study were guided by the PICOS strategy. The population/participants (P) consisted of patients with anterior or posterior restorations. The intervention (I) analyzed was direct restoration with single-shade RBC, and the comparator (C) was multi-shade RBC. The evaluated outcome (O) was color match. The study design (S) was randomized clinical trial.

The study inclusion criteria were as follows: (1) randomized clinical trial; (2) evaluation of the color match of single-shade RBCs in direct restorations; (3) use of the United States Public Health Service (USPHS) criteria and World Dental Federation (FDI) criteria to evaluate the clinical color match of the RBCs; and (4) use of multi-shade RBCs as comparative material. The exclusion criteria were as follows: (1) studies evaluating color matching with other methods; (2) in vitro studies; (3) studies using experimental single-shade RBCs; (4) unpublished information in the scientific literature; (5) studies for which the full text was not available; and (6) retrospective studies.

### 2.3. Information Sources and Search Strategy

The PubMed, Embase, Web of Science, Scopus, and Cochrane Library databases were searched for clinical studies evaluating the color stability of single-shade RBCs in direct restorations in April 2024. The following Medical Subject Headings (MESH) or text words were used: single-shade composite, monoshade universal composite, monochromatic composite, permanent dental restoration, permanent dental filling, randomized controlled trials as topic, randomized clinical trial, clinical trial, clinical data, clinical studies as topic, medical trial, intervention study, intervention trial, interventional study, and interventional trial. The following search was performed across all databases: [(“single-shade composite” OR “mono-shade universal composite” OR “monochromatic composite”) AND (“restorations, permanent dental” OR “restoration permanent dental” OR “dental permanent filling” OR “dental permanent fillings”)] AND [(“Randomized Controlled Trials as Topic” OR “Clinical Trials, Randomized” OR “Trials, Randomized Clinical” OR “Controlled Clinical Trials, Randomized” OR “Randomized Controlled Trial” OR “clinical trial” OR “clinical data” OR “clinical studies as topic” OR “medical trial” OR “intervention study” OR “intervention studies” OR “intervention trial” OR “interventional studies” OR “interventional study” OR “interventional trial”)] (Table 1).

### 2.4. Selection Process

The studies were stored and systematically organized using an online program (Rayyan Free version, Qatar Computing Research Institute, Ar-Rayyan, Qatar). Duplicates were first removed, and then the titles and abstracts were read to determine whether the studies met the predefined criteria. The selection process was carried out independently by two authors (CFCL and SBM) who were previously calibrated, and discrepancies were discussed with a third author (MAJRM). The calibration process facilitated by the two authors (CFCL and SBM) involved the joint reading and assessment of 10 articles to ensure congruence in the interpretation and application of the selection criteria. This collaborative review promoted a consistent approach to article selection based on predetermined criteria. Any disagreements that arose during independent selection were resolved through discussion and consensus with the third author (MAJRM). Eligible articles were carefully read, and their data were carefully extracted.

### 2.5. Data Collection Process

Two authors (CFCL and SBM), who were previously calibrated, performed the data extraction using a guide table covering the main methodological characteristics of the studies. Key data included author/year, RBCs used, number of subjects and age range, number of restored teeth, type of restored tooth, finishing and polishing protocol, light curing unit, type of restoration, follow-up period, analysis criteria, and conclusion.

### 2.6. Assessment of Study Risk of Bias

The included trials were assessed for risk of bias by two authors (CFCL and SBM), who had been previously calibrated. The assessment used the Cochrane Risk of Bias for Randomized Trials version 2 (RoB 2) tool, which includes domains assessing bias related to the randomization process, deviations from intended interventions, missing outcome data, measurement of outcomes, and selection of reported outcomes. Each domain is accompanied by signaling questions designed to systematically request relevant information for bias assessment, with responses categorized as yes, probably yes, probably no, no, or no information. Following the completion of the signaling questions, a risk of bias judgment was made, which was categorized as low risk of bias, some concerns, or high risk of bias. The RoB 2 tool contains algorithms that connect responses to signaling questions with suggested risk of bias assessments for each domain. In cases of disagreement between the two assessors, a third assessor was consulted to reach a consensus (MAJRM).

### 2.7. Effect Measures and Synthesis Methods

The meta-analysis was performed using a random-effects model. Review Manager version 5.4 (Review Manager 5.4, The Cochrane Collaboration) software was used to calculate the risk difference with a 95% confidence interval. The data were dichotomized for this analysis. Acceptable restorations were those that received the Alpha and Bravo scores for the USPHS criteria and scores of 1, 2, and 3 for the FDI criteria. The unacceptable restorations were those that received a Charlie score when the USPHS criteria were applied and those with scores of 4 and 5 when the FDI criteria were used to evaluate color stability. The unacceptable data were used in the meta-analysis. This analysis was performed with two subgroups according to the follow-up period: one considered the baseline (1 to 7 days), and the other considered after 12 months. Table 2 shows the USPHS and FDI grading criteria.

### 2.8. Certainty Assessment

The certainty of evidence for each outcome was evaluated utilizing the Grading of Recommendations, Assessment, Development, and Evaluation (GRADE) tool, accessible at http://www.gradeworkinggroup.org/, accessed on 17 April 2024. This tool evaluates the study design and considers factors such as risk of bias, imprecision, inconsistency, indirectness of evidence, and publication bias to potentially determine the quality of evidence. Each aspect is assessed as having “no limitation”, “serious limitations”, or “very serious limitations”, allowing for the classification of evidence quality as high, moderate, low, or very low. A lower quality indicates that the estimate may differ substantially from the true effect.

## 3. Results

### 3.1. Study Selection

**Figure 1:**
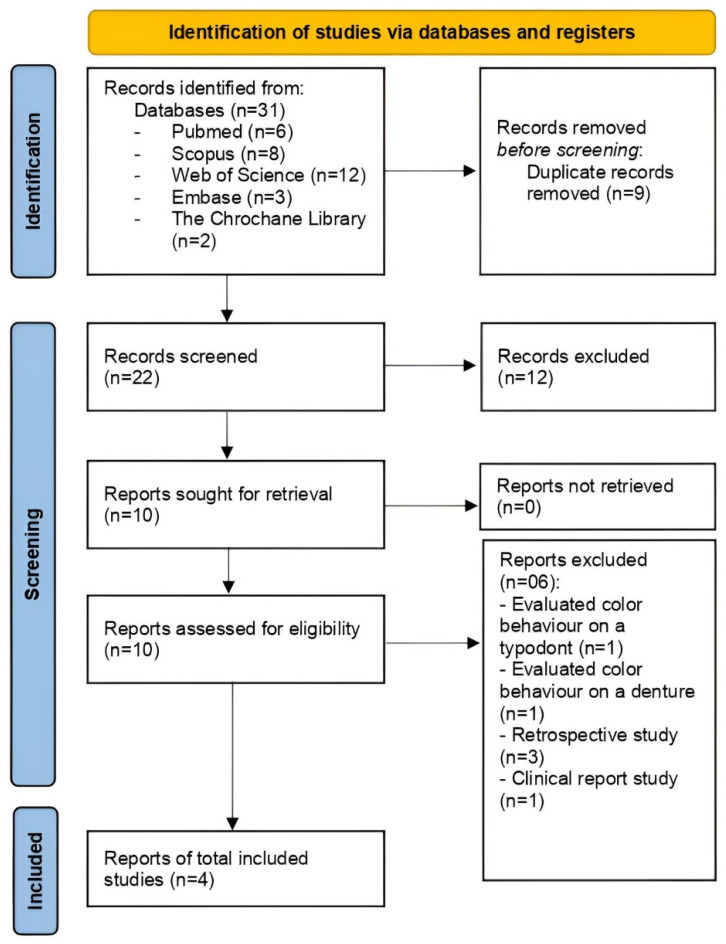
PRISMA flow diagram of the literature search and selection criteria.

A total of 31 studies were extracted from the databases in the search conducted in April 2024. After removing duplicates, 22 studies remained, and their titles and abstracts were analyzed according to the predefined inclusion and exclusion criteria. The full texts of 10 potentially eligible studies were read, four of which met the selection criteria (Figure 1). One study was excluded for assessing color behavior on a typodont [22], another for evaluating color on a denture [23], three studies were retrospective [14,24,25], and the last study was a clinical report study [26].

### 3.2. Study Characteristics

The characteristics of the studies are shown in Table 3. The included articles were published between 2022 and 2024. The number of participants ranged from 20 to 70. The age of the subjects ranged from 43 to 58 years, with follow-up periods ranging from 3 days to 1 year [1,19,26,27]. The countries where the trials were conducted were Egypt [1,28], Brazil [27], and India [19].

The total number of patients treated and teeth restored were 141 and 236, respectively. One study included class IV restorations [28], one study included full coronal esthetic restorations [19], one study included non-carious cervical lesions [27], and one study included occlusal restorations [1]. The restored dental elements included permanent anterior and posterior teeth and primary anterior teeth. Of the selected studies, only Miranda et al. [27] used the FDI criteria, and the others used the USPHS. The single-shade RBCs used were Filtek Universal [28], Omnichroma [1,19], and Admira Fusion X-tra [27]. The multi-shade RCBs used were Filtek z350 xt [28], Tetric N Ceram [1,19], and Admira Fusion [27]. All studies reported the use of absolute isolation. The light-curing units used were an Elipar S10 [26], a Bluephase meter II [27], and a Bluephase Style [1]. One study did not report the light-curing device used [19]. All studies cured for 20 s per increment.

Regarding the method of resin color selection, the polychromatic technique used in the study by Hashem, Kairy, and Shaalan [28] involved the application of a palatal layer of enamel resin, followed by subsequent layers of dentin and enamel resin. However, the color scale used was not reported. Miranda et al. [27] used a shade guide from the Admira Fusion kit for shade selection, using combinations of two or more shades when necessary. Zulekha et al. [19] reported color selection for the control group only but did not detail the methodology used. Anwar et al. [1] did not clarify the method of color selection. In terms of finishing and polishing techniques, Hashem, Kairy, and Shaalan [28] initially used yellow-coded diamond finishing stones, Soflex discs, Perio-Bur #831, rubber cups, flames, and wheel polishing tips. Miranda et al. [27] employed fine and extrafine #2200 diamond burs and OptraPol NG. Sulekha et al. [19] did not report the specific finishing and polishing technique used. Anwar et al. [1] used low-speed fine grit diamond finishing stones supplemented with EVE DIACOMP Plus Occuflex impregnated rubber cups and impregnated brushes, following a recommended sequence (coarse, medium, fine, and superfine).

### 3.3. Risk of Bias in Studies

**Figure 2:**
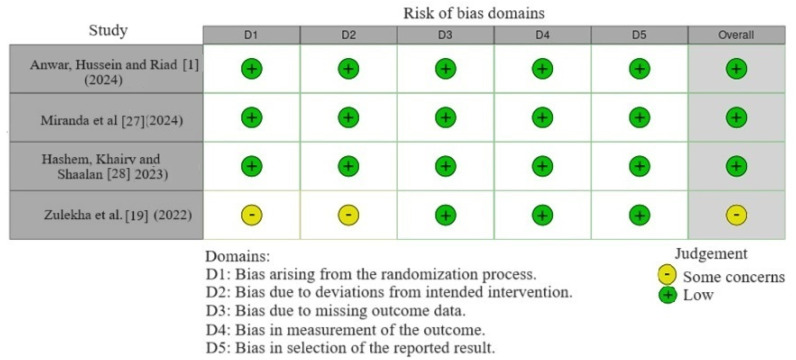
Risk of bias analysis for the randomized clinical trials. [1,19,27,28].

The RoB 2 tool was used for the randomized clinical trials. Randomization is a crucial component in clinical trials to ensure the validity of the results by minimizing selection bias. Zulekha et al. [19] did not describe the randomization process, which raises concerns about potential biases that could have influenced their findings. In contrast, Hashem, Khairy and Shalaan [28] and Anwar et al. [1] used the site “randomization.com”, and Miranda et al. [27] used the site “sealenvelope.com”, ensuring a more robust allocation of participants. Regarding the blinding of participants, examiners, and staff, Hashem, Khairy and Shalaan [28], and Miranda et al. [27] used opaque sealed envelopes. Anwar, Hussein and Riad [1] did not mention the method used for blinding, although they clarified that this precaution was taken. Zulekha et al. [19] presented incomplete data on allocation knowledge by outcome assessors and patients. Three [1,27,28] studies exhibited a low risk of bias, with all expected results and planned analyses documented, and one [19] exhibited some concerns regarding the blinding and allocation of participants/personnel/outcome assessors (Figure 2).

### 3.4. Results of Syntheses

**Figure 3:**
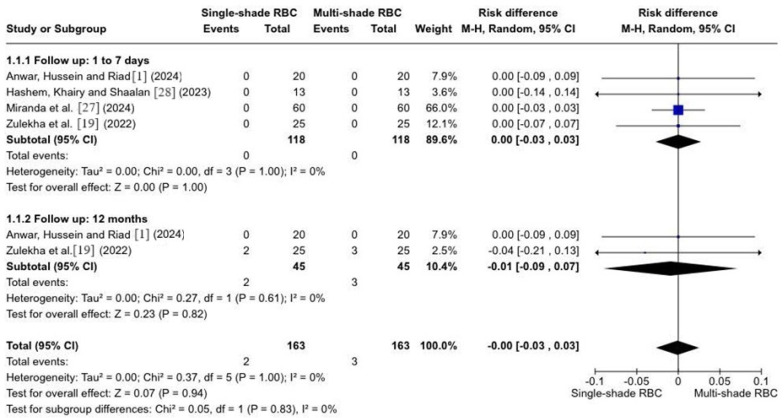
Forest plot of color behavior. [1,19,27,28].

For the meta-analysis, studies using the United States Public Health Service (USPHS) criteria were extracted for the proportion of restorations considered with worse scores (Charlie), and studies using the World Dental Federation (FDI) were extracted for restorations with equivalent worse scores (scores 4 and 5) according to the following criteria: color match. Four studies were included. No statistically significant differences (*p* > 0.05) were found between single-shade and multi-shade resins for 1 to 7 days (*p* = 1.0; RD = 0.00; 95% CI −0.03–0.03; I^2^ = 0%) or 12 months (*p* = 0.61; RD= −0.01; 95% CI −0.09, 0.07; I^2^ = 0%) (Figure 3).

### 3.5. Certainty of Evidence

Table 4 shows that the certainty of the evidence of color stability was low for all follow-up periods. Although the included studies had a low risk of bias, issues of indirectness and imprecision were deemed significant due to sample size limitations (fewer than 300 for dichotomous outcomes). Consequently, the certainty of evidence was reduced due to the small number of events and the small sample size.

## 4. Discussion

For direct restorations with RBCs, color selection is an important phase. Due to the characteristics of natural teeth, it is necessary to choose the right shade of composite to achieve esthetic procedures. The aim of this systematic review and meta-analysis was to investigate the shade performance of single-shade RBCs in direct restorations. This was achieved by evaluating restorations using clinical instruments such as the USPHS and FDI criteria, taking into account scores indicating failure (Charlie, score 4, and score 5). Two follow-up periods were analyzed: one ranging from 1 to 7 days and the other after 12 months. The first follow-up period allowed us to assess the color match of the restorations, while the second period provided information on the long-term clinical color stability of the RBCs examined. The results of the meta-analysis showed that single-shade RBCs performed similarly to multi-shade RBCs at both time points.

The single-shade RBCs used in the studies included in this systematic review were Filtek Universal [28], Omnichroma [1,2,3,4,5,6,7,8,9,10,11,12,13,14,15,16,17,18,19], and Admira Fusion X-tra [27]. Table 5 shows the composition of the single-shade RBCs. The Filtek Universal RBCs feature NaturalMatch technology and offer eight designer shades and an extra-white option that effectively covers the 19 VITA Classic and Bleach shades [29]. Omnichroma RBC features Smart Chromatic Technology, which makes it possible to reproduce the shades of the entire Vita shade range from A1 to D4 [30]. Admira Fusion X-tra is a single-shade bulk fill resin with nanohybrid particles and ORMOCER technology. Admira Fusion X-tra is a single-shade bulk-fill resin with only one shade, as the designer nanoparticles neither diffract nor refract light [31].

All single-shade RBCs tested showed comparable color matches to those of the multi-shade resins over the 1- to 7-day period. This is an important feature, as color is a key factor in smile preference for both dentists and patients. Errors in color selection can lead to patient dissatisfaction and the need for costly replacement restorations, putting healthy tooth structures at risk [11]. Filtek Universal has non-aggregated and non-aggregated 20 nm silica fillers, where these particles may contribute to the opacity that explains the acceptable shade matching potential found in this systematic review [28]. NaturalMatch Technology consists of nanoparticles, pigments, and low-stress monomers that contribute to the formation of “white lines” at the margins [29]. Uniform spherical Omnichroma RBCs measuring 260 nm are capable of transmitting red-to-yellow color when ambient light passes through the resin [23]. As the red-to-yellow color is produced, it blends with the surrounding tooth color in an additive color mixing process, maximizing the ability of Omnichroma to mimic the color of the dental element without the need for pigments [30], which is consistent with our results. Admira Fusion X-tra contains spherical and uniformly sized nanoparticles that prevent diffraction or refraction of light. These nanoparticles allow light to pass through them and reach the surface of the tooth. The light is then reflected to the human eye, influenced by the adjacent tooth color [31], justifying the results of this systematic review.

Studies show that the blending effect is increased by increasing the translucency, as this property allows the composite to mimic the shade of the neighboring dentin and enamel [32]. Filtek Universal RBCs contain nanoclusters, which may be responsible for translucency and light transmission [28]. The high translucency of Omnichroma is due to a specific combination of the type (uniform spherical particles) and fraction (79%) of the inorganic phase. The absence of colorants in Omnichroma reduces energy attenuation or loss in the material, which justifies its exceptional translucency [33]. The Admira Fusion X-tra shade is 45.56% more translucent than the dentin shade and 27.5% more translucent than the enamel shade [32]. These characteristics support the findings of this study regarding color matching. The translucency required to enhance color matching appears to be a concern for esthetic restorations [1]. Miranda et al. [27] reported that color matching was achievable regardless of the degree of dentin sclerosis. It is important to note that to achieve favorable color results with multi-shade resin restorations, the operator must be trained to use the incremental technique with different shades, which is not necessary when using single-shade resin [27].

The evaluation of the success of restorative therapy probably depends on the durability of the restorations [19]. One of the parameters associated with durability is color stability. All the single-shade RBCs investigated showed comparable color stability to that of the multi-shade resins during the 12-month follow-up period. Finishing and polishing procedures for resin composite restorations are essential steps to improve the appearance, color stability, and longevity of the restorations [28]. Almost all included studies mentioned the finishing and polishing techniques, except for Zulekha et al. [19], who did not mention whether this step was performed or how it was carried out. The RBCs studied have similar filler contents and contain nanospherical filler particles, which provide a smoother finish to the restoration and fewer spaces between the resin–filler interface, reducing bacterial adhesion and microleakage, and consequently minimizing staining over time [19]. Another factor that may be related to color stability is the size of the particles; small particles contribute to less staining and improved esthetic properties [26]. In a retrospective study, a single-shade resin-based composite (RBC) achieved a 100% acceptable color match after 4 years in previous restorations for diastema closure and contouring [14]. Color mismatch was the least common cause of restoration failure identified by Korkut et al. [26] in a retrospective study evaluating the performance of single-shade RBCs in Class IV cavities. Only one restoration had the lowest score after 4 years of follow-up [26].

It is important to highlight that long-term color stability is also influenced by the staining capability of the restorative material when it is in contact with foods and beverages containing dyes. The scoping review conducted by Paolone et al. [34] presented conflicting in vitro studies regarding the single-shade bulk-fill resin Admira Fusion X-tra. One study revealed that this single-shade resin exhibited a higher discoloration rate than conventional bulk-fill resin did, while another study revealed that it had less pigmentation after artificial aging. The negative behavior was likely attributed to insufficient integration between the resin matrix and the siloxane particles, allowing greater dye diffusion [34]. In contrast, the positive behavior was due to the conventional bulk-fill resin comparator having a higher organic matrix content [34]. In an in vitro study, Omnichroma resin also showed a high staining potential after storage challenges in tea and red wine, especially compared to a multi-shade resin. Omnichroma contains low molecular weight monomers, increasing water sorption [35]. Resins with pigments tend to have better color matching after staining, which also explains the behavior of the Filtek universal resin, which shows a greater predisposition to color changes compared to other studied multi-shade resins [36].

Based on the results of this study, single-shade RBCs exhibited similar color characteristics to multi-shade RBCs. These findings can help clinicians support the use of dental materials that mimic and harmonize with natural tooth shades, providing color stability. The majority of the studies included in this systematic review had a low risk of bias, but the certainty of the evidence was rated as low due to the small number of events and sample size, which may not have provided sufficient evidence. Only one study [19] showed some concerns about the blinding and allocation of participants/personnel/outcome assessors. The limitations of this study are related to the length of follow-up, which was only up to one year. At least a decade of observation is required to accurately assess differences in treatment efficacy, as restorative materials may have different failure rates over time [37]. Nevertheless, this topic remains relatively unexplored in the literature, mainly due to the lack of randomized clinical trial (RCT) studies.

Future studies including diverse age groups are also encouraged to better understand the clinical behavior of the color adjustment potential in both young and mature teeth, as they exhibit different optical characteristics. Several studies included in the meta-analysis used the USPHS criteria, which are known for their limited sensitivity in definitively assessing the success of a restorative procedure [38]. Conversely, the FDI criteria have been shown to be more sensitive and accurate than the USPHS standards in identifying subtle differences in clinical studies [39]. The conclusions drawn from this systematic review and meta-analysis should be treated with caution due to the limited availability of clinical studies. We advocate the conduct of additional randomized clinical trials to assess the clinical performance of these resins, particularly with longer follow-up periods, as initial evaluations tend to have fewer events. In particular, the high discoloration potential of single-shade resins can affect long-term clinical success [17].

## 5. Conclusions

This systematic review and meta-analysis revealed that the color match and color stability of direct restorations with single-shade RBCs were similar to those of direct restorations with multi-shade RBCs over a 12-month period. However, these results should be considered with caution due to the low certainty of evidence regarding small events and the small sample size of the included studies.

## Figures and Tables

**Table 1:** Search strategy.

Database	Search Strategy
**Pubmed**	(Single-shade [All Fields] AND (composite [All Fields] OR composites [All Fields])) OR (monoshade [All Fields] AND universal [All Fields] AND (composite [All Fields] OR composites [All Fields])) OR ((monochromatic [All Fields]) AND (composite [All Fields] OR composites [All Fields])) AND (dental restoration, permanent [MeSH Terms] OR dental restoration, permanent [MeSH Terms] OR dental restoration, permanent [MeSH Terms] OR dental restoration, permanent [MeSH Terms]) AND (Randomized controlled trials as topic [MeSH Terms] OR randomized controlled trials as topic [All Fields] OR clinical trials randomized [All Fields] OR clinical trials randomized [All Fields] OR trials randomized clinical [All Fields] OR trials randomized clinical [All Fields] OR controlled clinical trials randomized [All Fields] OR controlled clinical trials randomized [All Fields] OR randomized controlled trial [All Fields] OR randomized controlled trial [All Fields] OR clinical trial [All Fields] OR (clinical [All Fields] AND data [All fields]) OR clinical studies as topic [MeSH Terms] OR clinical studies as topic [All Fields] OR (Medical [All Fields] AND trial [All Fields]) OR intervention study [All Fields] OR (intervention [All Fields] AND trial [All Fields]) OR (interventional [All Fields] AND trial [All Fields]) OR (Interventional [All Fields] and Study [All Fields]) OR intervention studies [All Fields])
**Embase**	(‘Single-shade composite’ OR ‘monoshade universal composite’ OR ‘monochromatic composite’) AND (‘dental restoration’/exp OR ‘dental restoration’ OR ‘Restorations, Permanent Dental’ OR ‘Restoration, Permanent Dental’ OR ‘Dental Permanent Fillings’ OR ‘Dental Permanent Filling’) AND (‘Randomized Controlled Trials as Topic’ OR ‘Clinical Trials, Randomized’ OR ‘Trials, Randomized Clinical’ OR ‘Controlled Clinical Trials, Randomized’ OR ‘Randomized Controlled Trial’ OR ‘clinical trial’ OR ‘clinical data’ OR ‘clinical studies as topic’ OR ‘medical trial’ OR ‘intervention study’ OR ‘intervention studies’ OR ‘intervention trial’ OR ‘interventional studies’ OR ‘interventional study’ OR ‘interventional trial’
**Web of Science**	TS = (“Single-shade composite” OR “monoshade universal composite” OR “monochromatic composite”) AND TS = (“Restorations, Permanent Dental” OR “Restoration, Permanent Dental” OR “Dental Permanent Fillings” OR “Dental Permanent Filling”) AND TS = (“Randomized Controlled Trials as Topic) OR (Clinical Trials, Randomized” OR (Trials, Randomized Clinical) OR (Controlled Clinical Trials, Randomized) OR (Randomized Controlled Trial) OR (clinical trial) OR (clinical data) OR (clinical studies as topic) OR (medical trial) OR (intervention study) OR (intervention studies) OR (intervention trial) OR (interventional studies) OR (interventional study) OR (interventional trial)
**The Chrochane Library**	(Single-shade composite) OR (monoshade universal composite) OR (monochromatic composite) AND (Restorations, Permanent Dental) OR (Restoration, Permanent Dental) OR (Dental Permanent Fillings) OR (Dental Permanent Filling) AND ((Randomized Controlled Trials as Topic) OR (Clinical Trials, Randomized) OR (Trials, Randomized Clinical) OR (Controlled Clinical Trials, Randomized) OR (Randomized Controlled Trial) OR (clinical trial) OR (clinical data) OR (clinical studies as topic) OR (medical trial) OR (intervention study) OR (intervention studies) OR (intervention trial) OR (interventional studies) OR (interventional study) OR (interventional trial)
**Scopus**	‘Restorations, AND permanent AND dental’ OR ‘restoration, AND permanent AND dental’ OR ‘dental AND permanent AND fillings’ OR ‘dental AND permanent AND filling’ AND ‘Single-shade and composite’ OR ‘monoshade and universal and composite’ OR ‘monochromatic and composite’ AND ‘Randomized Controlled Trials as Topic’ OR ‘Clinical Trials, Randomized’ OR ‘Trials, Randomized Clinical’ OR ‘Controlled Clinical Trials, Randomized’ OR ‘Randomized Controlled Trial’ OR ‘clinical trial’ OR ‘clinical data’ OR ‘clinical studies as topic’ OR ‘medical trial’ OR ‘intervention study’ OR ‘intervention studies’ OR ‘intervention trial’ OR interventional studies’ OR ‘interventional study’ OR ‘interventional trial’

**Table 2:** USPHS and FDI criteria for color behavior.

**The USPHS Criteria**
Color match
Alpha (A): The restoration matches the adjacent tooth tissue in color, shade, or translucency.
Bravo (B): There is a slight mismatch in color, shade, or translucency, but within the normal range of adjacent tooth structure.
Charlie (C): There is a slight mismatch in color, shade, or translucency, but outside of the normal range of adjacent tooth structure.
**FDI Criteria**
Color stability or translucency
Score 1: Good coloration and translucency compared to neighboring teeth.
Score 2: Minimal color and translucency deviation.
Score 3: Clear deviation, but without affecting esthetics.
Score 4: Localized clinical deviation that can be corrected by repair.
Score 5: Unacceptable, replacement necessary.

**Table 3:** Data extracted.

Author, Year	Study Design	RBCs	No. of Subjects (Mean Age)	No. of Rest.	Tooth	Finish and Polish	Light Curing	Type of Rest.	Follow-Up	Analysis Criteria	Conclusions
Hashem, Khairy and Shaalan [28] (2022)	RCT	Filtek universal Filtek Z3250 XT	26 (13–30) years	26	Permanent incisors	Yellow-coded diamond finishing stones, Soflex discs, perio-bur #831, and rubber cup, flame, and wheel polishing tips	Elipar S10—20 s	Class IV	3 days	USPHS	Single-resin RBC showed satisfactory shade matching potential compared to polychromatic RBCs.
Zulekha et al. [19] (2022)	RCT	Omnichroma Tetric N Ceram	25 (3–5 years)	50	Primary anterior teeth	NM	NM	Full coronal esthetic	12 months	USPHS	Single-shade RBC performed similarly to multi-shade in terms of the color match and color stability for both 6- and 12-month intervals.
Miranda et al. [27] (2024)	RCT	Admira Fusion Admira Fusion X-tra	70 (40–58 years)	120	Anterior or posterior	Fine and extrafine #2200 diamond burs along with OptraPol NG	Bluephase meter II—20 s	NCCL	7 days	FDI	The single-shade RBC used achieves the same color match compared to a multi-shade composite resin after 7.
Anwar, Hussein and Riad [1] (2024)	RCT	Omnichroma Tetric N Ceram	20 (20–45 years)	40	Molar or premolar	Low-speed fine-grit diamond finishing stones, EVE DIACOMP Plus Occuflex-impregnated rubber cups and impregnated brushes	Bluephase Style—20 s.	Oclusal cavities	12 months	USPHS	Single-shade RBC exhibited comparable performance to a multi-shade RBC regarding color match and color stability.

Legend: RCT = randomized clinical trial; RBCs = resin-based composites; No = number; Rest = restorations; NM = not mentioned; NCCL = non-carious cervical lesion; FDI = World Dental Federation; USPHS = United States Public Health Service.

**Table 4:** Evidence certainty assessment with grading of recommendations, assessment, development, and evaluation (GRADE).

Parameter	No. of Studies	Study Design	Risk of Bias	Inconsistency	Indirectness	Imprecision	Other Considerations	Certainty
Color stability (1 to 7 days)	4	RCT	Not serious ^a^	Not serious ^b^	Serious ^c^	Serious ^d^	None	⊕⊕OO Low
Color stability (12 months)	4	RCT	Not serious ^a^	Not serious ^b^	Serious ^c^	Serious ^d^	None	⊕⊕OO Low

GRADE Working Group grades of evidence. ⊕⊕⊕⊕ High quality: Further research is very unlikely to change our confidence in the estimate of effect. ⊕⊕OO Moderate quality: Further research is likely to have an important impact on our confidence in the estimate of effect and may change the estimate. ⊕⊕OO Low quality: Further research is very likely to have an important impact on our confidence in the estimate of effect and is likely to change the estimate. ⊕OOO Very low quality: We are very uncertain about the estimate. RCT: randomized clinical trial. ^a^ All included studies presented a low risk of bias, and one study reported some concerns about the blinding and allocation of participants/personnel/outcome assessors. ^b^ There was no substantial heterogeneity. ^c^ Indirectness judged based on population, intervention, comparison, and outcome across studies, where the population did not present sufficient direct evidence. ^d^ The evidence was downgraded because the number of events and sample size were considered small.

**Table 5:** Materials’ specifications and composition of single-shade RBCs.

Single-Shade RBC	Manufacturer	Composition
Filtek Universal	3 M ESPE, St. Paul, MN, USA	Fillers are a combination of a non-agglomerated/non-aggregated 20 nm silica filler, a non-agglomerated/non-aggregated 4 to 11 nm zirconia filler, an aggregated zirconia/silica cluster filler (comprised of 20 nm silica and 4 to 11 nm zirconia particles), and a ytterbium trifluoride filler. The inorganic filler loading is about 76.5% by weight (58.4% by volume). Matrix: AUDMA, AFM, diurethane-DMA, and 1,12-dodecane-DMA.
Omnichroma	Tokuyama Dental, Tokio, Japan	Matrix: TEGDMA, UDMA, Dibutyl hydroxyl toluene and UV absorber, Mequinol. Filler system: SiO2, ZrO2 (68 vol.—%; 79 wt%; 0.2–0.4 μm)
Admira Fusion X-tra	Voco GmbH, Cuxhaven, Germany	Organically modified ceramic ORMOCER. Matrix: aromatic and aliphatic dimethacrylates, methacrylatefunctionalized polysiloxane. Filler: barium aluminum borosilicate glass ceramic filler (median: 1 mm) and silicon dioxide nanoparticles (0.02 to 0.04 μm). Filler 84 wt%. Camphorquinone Pigments: ironoxide and titanium dioxide

Legend: AUDMA = aromatic urethane dimethacrylate; AFM = addition-fragmentation monomer; TEGDMA = tetraethylene glycol dimethacrylate; UDMA = urethane dimethacrylate.

## Data Availability

All the data are available within the manuscript.
